# Proactive concussion prediction using symmetry-aware multiscale explainable hybrid deep learning and multimodal data fusion

**DOI:** 10.1038/s41598-026-54871-9

**Published:** 2026-08-12

**Authors:** Akinbowale Nathaniel Babatunde, Damilare Peter Oyinloye, Roseline Oluwaseun Ogundokun, Folasade Abimbola Aluko, Ayodele Babatunde, Akeem Femi Kadri, Shuaib Babatunde Mohammed, Damilola Popoola, Joseph Bamidele Awotunde, Rotimi-Williams Bello

**Affiliations:** 1 Department of Computer Science, Kwara State University, Malete, Nigeria; 2 Department of Computer Science, Norwegian University of Science and Technology, Trondheim, Norway; 3 Department of Multimedia Engineering, Kaunas University of Technology, Kaunas, Lithuania; 4 Department of Computer Science, Redeemer’s University Ede, Ede, Osun Nigeria; 5 Department of Computer Systems Engineering, Tshwane University of Technology, Pretoria, South Africa; 6 Department of Mathematics and Computing, Thomas Adewumi, Oko, Nigeria; 7 Information and Engineering Department, Fuzhou Melbourne Polytechnic, Fuzhou, China; 8 Department of Statistics, Faculty of Physical Sciences, University of Ilorin, Ilorin, 240003 Nigeria; 9 Department of Computer Engineering, Faculty of Engineering and Architecture, Recep Tayyip Erdoðan Üniversitesi, Zihni Derin Yerleþkesi, Fener, 53100 Merkez/Rize, Türkiye

**Keywords:** Concussion prediction, Explainable AI, Transformer network, Multimodal fusion, Symmetry-aware learning, EEG analysis, Computational biology and bioinformatics, Engineering, Mathematics and computing, Neurology, Neuroscience

## Abstract

Diagnosis of concussion has been hampered by its subtle and late symptoms, the reliance on subjective judgment, and the lack of real-time multimodal data integration. The developed computational models are generally black boxes and lack the robustness required for safety-critical scenarios. We introduce a symmetry-aware, multiscale, explainable, hybrid deep learning framework for early prediction of concussion risk. The system effectively combines kinematic sensor data, EEG, contextual information, and synchronized video using separate modality-specific feature extractors and a multimodal fusion layer that utilizes attention-based multimodality fusion. The framework is evaluated using subject-independent 5-fold cross-validation to evaluate its generalizability. The proposed framework incorporates explainability with SHAP value analysis and attention visualizations. The proposed system achieves an accuracy of 0.91, a macro F1-score of 0.90, and an AUC of 0.95, outperforming baselines that rely on single modalities and traditional multimodal integration. High concussion risk events are recalled with an F1-score of 0.89, and moderate concussion events are predicted with an F1-score of 0.87 to capture subtle injury characteristics. We showed that symmetry-aware multiscale fusion, combined with an explainable decision support system, provides a useful tool for concussion risk prediction. This framework holds promise for clinical decision support, although further clinical trials with larger, more diverse populations are necessary to establish its reliability for real-world implementation.

## Introduction

Concussion has been an ongoing problem in public health, especially within the context of physically demanding and competitive sport activities, as well as strenuous operational conditions where high-impact forces can repeatedly be applied to the head. In fact, concussion and minor TBI may result in ambiguous and variable symptoms that can interfere with an immediate diagnosis and increase the probability of long-term damage if such injuries are not addressed early^1,2^. Rapid development in AI and intelligent sensing has opened a promising avenue for anticipatory concussion risk assessment, in addition to reactive post-impact clinical testing^3,4^. In terms of application, this study would be relevant to sports medicine, neuro-trauma monitoring, and intelligent healthcare systems, where early identification of concussion helps mitigate potential long-term neuro-psychological consequences. Advanced helmets with integrated sensing capabilities are capable of continuously logging kinematics of the head, forces being applied, and dynamic behavior during sport or intense physical work^5,6^, and flexible morphologically adaptive sensing can further enable reliable detection of head impacts under complex, dynamic operation environments^7^. The presence of sensor-equipped cloud systems enables distributed, near real-time acquisition, and processing of biomechanical and physiological signals^8^.

Despite these advancements, several factors remain that impede the accuracy of current concussion prediction algorithms. Using only biomechanical impact thresholds does not sufficiently capture the nature of neurophysiological responses to injury and thus is not sensitive to moderate-risk concussions. In fact, studies utilizing neuroimaging and neural signals show evidence of structural and functional brain abnormalities, even in the absence of immediately identifiable signs of injury, underscoring the value of objective neurophysiological biomarkers for diagnosis^9^. Alone, however, neurophysiological models are also prone to signal noise, context independence, and loss of robustness outside the laboratory.

Therefore, multimodal learning has become one of the most promising and key techniques for enhancing prediction reliability by fusing physiological, biomechanical, and contextual information. Several previous studies in biomedical and clinical prediction have clearly demonstrated that multimodal heterogeneity can enhance classification accuracy and model generalisation more than any single modality model^10,11^. In the application of deep learning, attention-based and hybrid deep learning models enable us to achieve both greater adaptability and robustness by adaptively focusing on informative modalities across varying data-quality levels. Meanwhile, some applications using convolutional neural networks to perform real-time monitoring of helmets and safety during athletic activities have also shown potential for developing an intelligent prediction system operating in dynamic operational contexts^12^. Motivated by these developments, this study proposes an attention-driven multimodal deep learning framework that integrates kinematic sensor data, neurophysiological signals, and contextual information to predict concussion risk. Adaptive fusion mechanisms and explainability-oriented learning strategies are incorporated to improve predictive robustness, interpretability, and the reliability of concussion-risk assessment in real-world sports and operational environments.

## Review of related works

Existing concussion prediction research increasingly recognizes mild traumatic brain injury as a multidimensional problem requiring integration of biomechanical, neurophysiological, and contextual information for reliable assessment. While traditional clinical measures have some utility in neuro screening, they are often insufficiently sensitive to subtle or delayed concussive signs, especially when recurrent events occur^1,2,13^. Such shortfalls have led to increased interest in implementing artificial intelligence and intelligent sensor technologies to enable predictive assessment of concussion risk. Recent research has shown that sensors capable of recording head kinematics, impacts, and force distributions, when implemented as wearable sensing systems in high-risk sporting or operating situations, are a step in this direction^6,14^. The development of flexible and embedded sensor technology enables greater ecological validity and continuous, long-term monitoring in real-world settings^7^. Direct evidence of concussive changes in brain network activity, provided by measures such as EEG and neural imaging, offers a complementary view of these events^15–17^. Similarly, clinical research also suggests a correlation between tissue strain and subsequent neurological abnormalities^18,19^.

In machine learning and deep learning, unimodal systems have evolved into multimodal fusion architectures that incorporate heterogeneous physiological and contextual data. CNN-, RNN-, LSTM-, and Transformer-based models have been extensively explored for concussion prediction and other related biomedical classification problems. CNNs are effective for learning spatial features from sensor or imaging data, while recurrent and LSTM-based models facilitate temporal sequence learning from physiological signals. Moreover, transformer- and attention-based architectures contribute to effective multimodal learning by adaptively weighting informative modalities in the presence of noise or incompleteness^11,20^. Many multimodal fusion studies have reported superior predictive performance compared to single-modality approaches^21,22^. Other relevant research in smart health systems, behaviour analytics, oncology, and biomedical imaging also demonstrates the extensibility of multimodal deep learning across various medical applications^4,23–25^.

Intelligent healthcare prediction systems have been greatly improved by combining biomechanics, neurophysiology, graph intelligence, and multimodal deep learning research to support explainable biomedical analytics. For example, Zhan et al.^26^ demonstrated the need for biomechanical accuracy when calculating a strain rate in relation to traumatic brain injury through developing computational frameworks for describing these structures; meanwhile, Zhang et al.^27^ and Chen et al.^28^ explored how different design techniques within deep learning architectures could lead to greater accuracy in identifying neurological activity associated with emotional states from EEGs. Additionally, Gan^29,30^ established topological algorithms based on graph representation theory to enhance the capabilities of large-scale graph intelligence and to provide new ways to perform computationally efficient relational operations, thereby directly facilitating effective multimodal data fusion solutions and assisting with contextual reasoning processes.

Research using advanced multimodal measurements and analyses will continue to highlight the value of multivariate biological data for improving our understanding of disease and increasing the reliability of predictions in healthcare and clinical practice^31,32^. For example, Wang et al.^33^ demonstrated that the use of advanced convolutional neural network (CNN) architectures can improve medical entity recognition performance in complex healthcare environments. Recent work in cognitive neuroscience^34^ supports the importance of EEG-informed decision modelling, while Kang et al.^35^ described how precisely engineered intelligent systems can enhance biomedical sensing capabilities and reduce signal response times. In addition, the advanced graph-based learning methods proposed by Zhang et al.^36^ and Lu et al.^37^ will continue to underscore the importance of knowledge-aware representation learning and context-based link prediction for complex biomedical reasoning tasks. In summary, all these studies support the growing connection between explainable artificial intelligence (AI), multivariate fusion, graph learning, EEG analytics, and deep neural network architectures to create robust, interpretable, and clinically meaningful intelligent healthcare systems and provide solid theoretical and methodological support for the concussion prediction framework proposed in this study.

Despite these advances, several limitations remain evident in current literature. Most existing multimodal systems rely heavily on delayed fusion schemes, thereby compromising real-time applicability in live sporting and operational settings. Most existing deep learning methods are black boxes, which lowers physician confidence and practical reliability. Also, cross-population, sensor-configuration, and operational-condition generalisation remain problematic due to limited training datasets and domain-specific assumptions. Similarly, low computational feasibility and robustness to missing modalities, along with few evaluations under real conditions, hinder real-world applications^3,38^. Representative studies and relevant contributions/limitations were provided in Table 1. The comparative analysis in Table 1 highlights persistent gaps in interpretability, real-time adaptability, multimodal robustness, and concussion-specific validation.

**Table 1:** Summary of related theoretical models.

Author (year)	Model	Algorithm	Contribution	Gaps
Abrol et al.,^10^	Multimodal fusion model	Deep neural networks	Effective fusion of dynamic connectivity features	Limited interpretability
Asani et al.,^21^	BioscoreNet	Multimodal self-attention	Sensor-based TBI detection using attention fusion	Smartphone-limited sensing
Liu et al.,^11^	Attention-based fusion	Multimodal attention	Robust prediction under missing modalities	Not concussion-specific
Nishizawa et al.,^39^	Attention fusion model	Multimodal deep learning	Improved interpretability in clinical prediction	Limited neurotrauma validation
Tran,^38^	Uncertainty-aware model	Bayesian deep learning	Predictive uncertainty modeling	Limited multimodal integration
Ghonge et al.,^40^	Cycle-aware model	Hybrid deep learning	Physiological variability adaptation	Not applied to concussion
Babatunde,^41^	CNN-based classifier	DenseNet	Improved medical image classification	No multimodal fusion
Babatunde,^42^	Transfer learning model	MobileNet	Enhanced computational efficiency	Single-domain focus
Mazumder,^22^	Multimodal DL model	Hybrid deep learning	Pediatric mTBI detection using multimodal data	Limited real-time deployment
Mahootiha,^23^	Multimodal risk model	Deep neural networks	Improved recurrence risk prediction	Oncology-specific application
Ye et al.,^20^	Hybrid fusion framework	Multimodal NLP + DL	Enhanced clinical prediction models	Limited sensor integration

From the literature reviewed, we highlight three key research gaps:

1. Limited real-time multimodal integration under dynamic operational settings.
2. Poor interpretability and explainability of current deep learning methods.
3. Limited generalization over populations, sensing devices and deployment conditions.

**Fig. 1:**
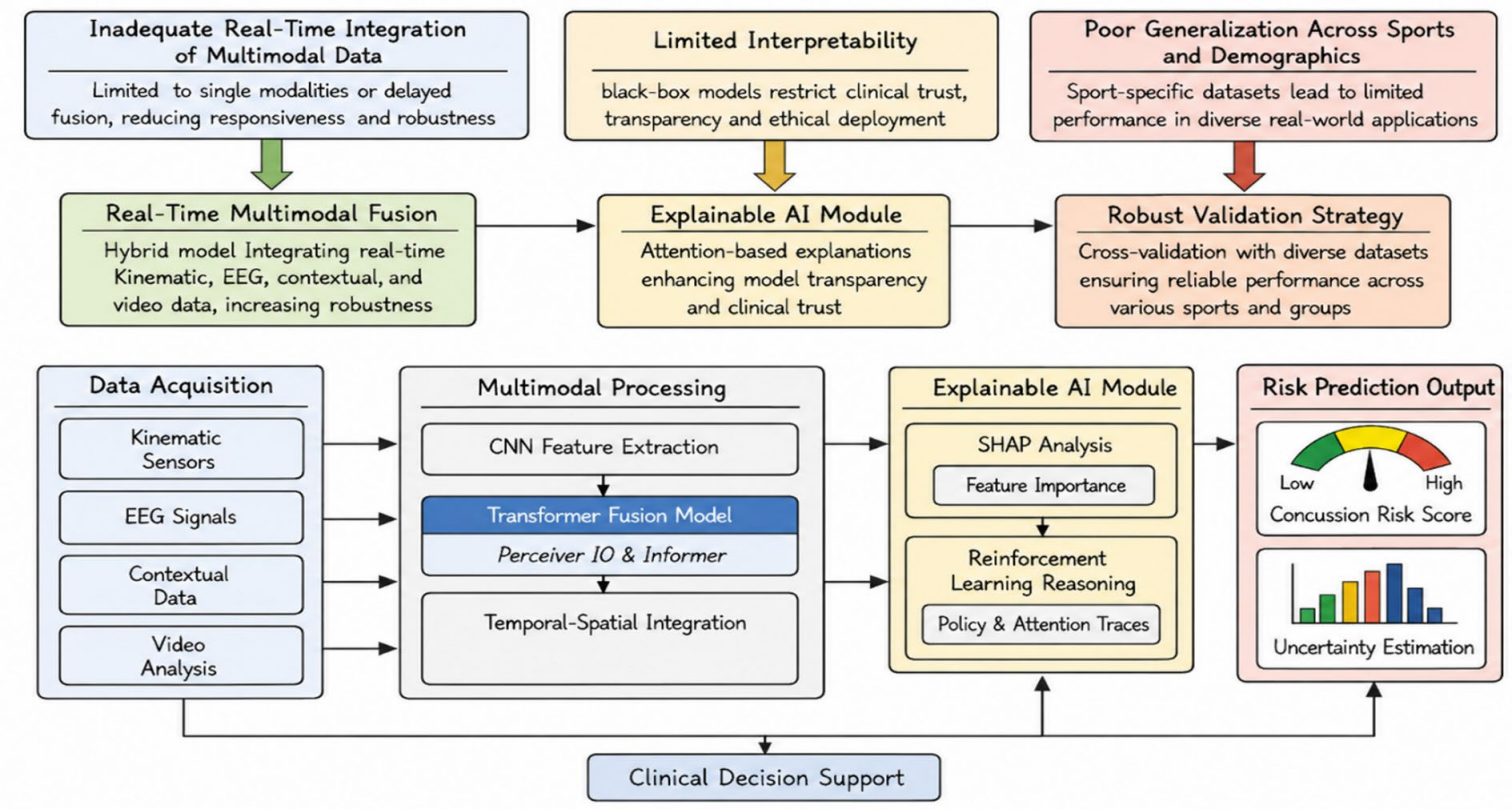
Mapping of identified gaps in existing concussion prediction systems to the components of the proposed attention-weighted multimodal deep learning framework.

Figure 1 illustrates the linkage between the above research gaps and the proposed framework components; attention-based multimodal fusion, explainable AI techniques, and generalization-aware validation mechanisms work together to overcome such challenges in real-time performance, robustness, and deployability.

## Methodology

### System overview

**Fig. 2:**
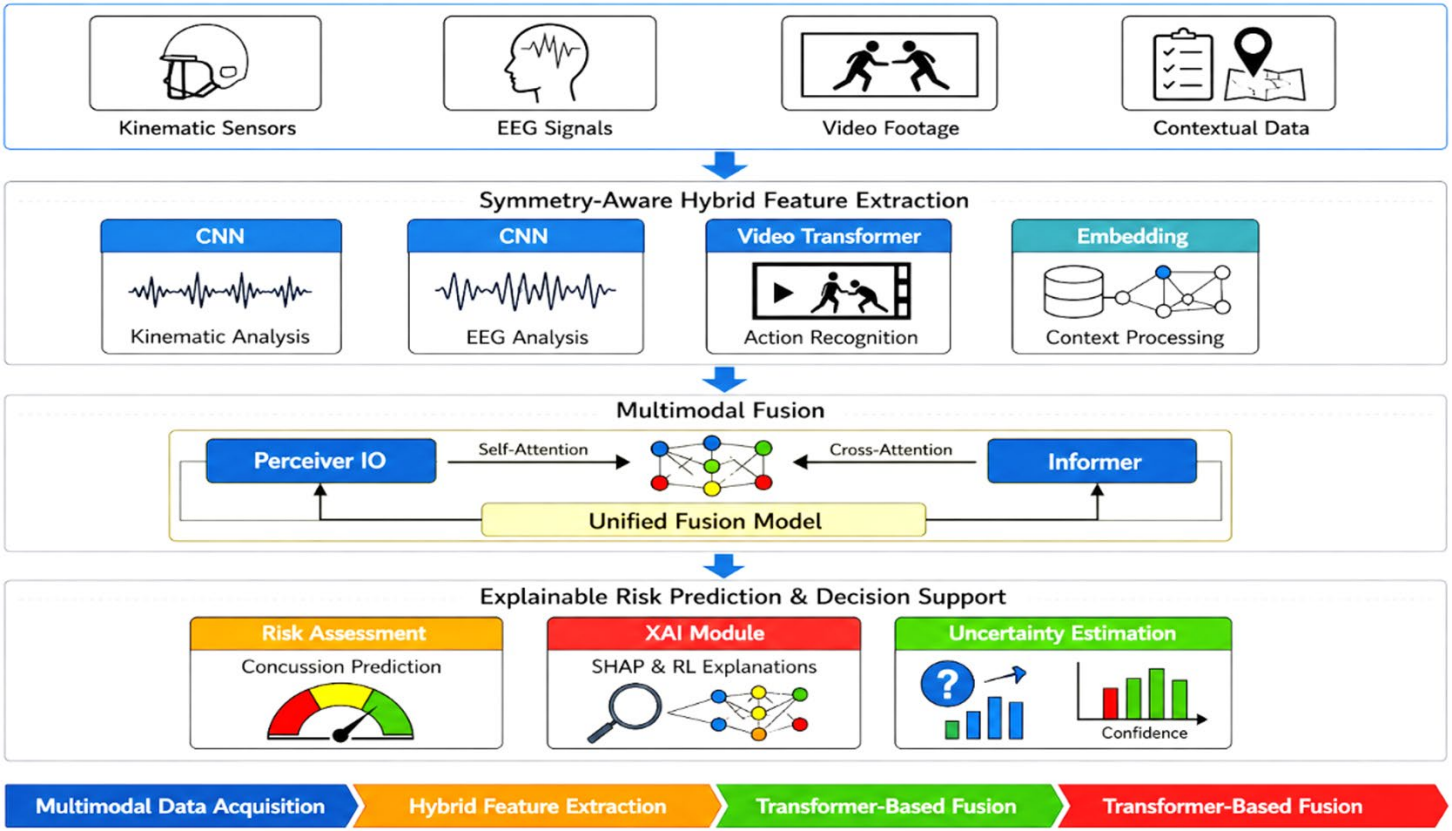
Symmetry-aware multiscale XAI-Hybrid mTBI framework.

The symmetry-aware multiscale XAI-Hybrid mTBI proposed herein can be described as a tiered end-to-end architecture for anticipatory and real-time mTBI risk prediction, prioritizing not only predictive accuracy and resilience but also clinical interpretability. The proposed framework combines multiple sensing modalities, hybrid deep learning approaches, and XAI within a unified processing workflow to build trust in decisions in safety-critical contexts such as contact sports and military settings. The architecture consists of 4 fundamental layers, depicted in Fig. 2: Multimodal Data Acquisition Layer, Symmetry-aware Hybrid Feature Extraction and Representation Learning Layer, Transformer-based Multimodal Fusion Layer, and Explainable Risk Prediction and Clinical Decision Support Layer. This stratification clearly illustrates how the performance-centric deep learning components are integrated with XAI elements to yield an auditable and medically interpretable prediction.

In the multimodal data acquisition layer, various asynchronous data streams are continuously captured from kinematic sensors, EEG headsets, environmental context streams, and situational video systems. The synergistic combination of information contributes to different views of a possible concussion event: mechanical aspects of the impact, neural mechanisms of the collision and/or reaction, the situation in which it occurs and the visible nature of the crash event. Timings of signals across shared impact regions are synchronized to permit cross modal learning in a unified manner, while avoiding potential inaccuracies due to misalignments. These temporalizations are useful for symmetry aware analysis.

To process the different modalities with architectures suited to their scale and inherent structure, the symmetry-aware hybrid feature extraction layer applies modality-specific learning structures to each data source. Specifically, CNNs process kinematic and EEG signals, which are high-dimensional, noisy, and temporally structured, to learn invariant and asymmetric local and multi-scale temporal patterns that correspond to dynamics associated with impact and neural perturbation effects. Short video clips are processed with space-time Transformer encoders to model global spatiotemporal relationships and to capture symmetric/asymmetric motion, body changes, collision angles, and interactions. Contextual metadata are converted into embedding layers while retaining their semantic structure and sequence dependency, to capture population-level and contextual information on a coarser semantic scale. These parallel, modality-specific learning methods will allow each source of information to be integrated appropriately without forcing them into a single learning pathway.

The multimodal fusion is central to the framework. The transformer-based fusion modules, such as Perceiver IO and Informer, combine the modality-specific embeddings via self-attention and cross-attention mechanisms. It enables learning higher-order interdependencies among biomechanical forces, neural disturbances, contextual risks, and visual features at both temporal and semantic scales. To achieve symmetry-aware integration by amplifying invariant cross-modal relations and mitigating asymmetric deviations reflecting injury mechanisms, the attention weights dynamically adapt on a per-event basis.

The last layer is for explainable prediction and clinical support. It involves a fully connected classification head that outputs a probabilistic concussion risk score and an integrated explainable AI module that provides transparent explanations for each prediction. Feature-level and modality-level attribution is achieved using Shapley Additive Explanations, and sequence decision logic is visualised via reinforcement-learning-based reasoning traces. Furthermore, uncertainty estimation enhances prediction confidence by attaching a confidence score to each risk estimate, enabling clinicians and practitioners to gauge the confidence of each automated decision. Collectively, this layered system architecture demonstrates how symmetry-aware multiscale hybrid deep learning and explainable AI can be cohesively integrated within a single framework, addressing the limitations of black-box concussion prediction models by combining strong predictive performance with interpretability, traceability, and clinical relevance.

### Multimodal data acquisition

Multimodal data collection is the foundation of the presented XAI-Hybrid mTBI framework and can capture a richer, less biased picture of head-impact events. The multimodal data collected in this experiment are from a public, readily available dataset of heterogeneous data streams that are each reflective of a different, yet complementary aspect of head impact, including mechanical impact forces, neurophysiological behavior, risk factors, and situational context, thereby decreasing reliance on subjective symptom reports, increasing learning/prediction integrity. The first stream of data includes kinematic data previously collected on helmet- or wearable sensors using inertial measurement units (IMUs), comprising linear acceleration, angular velocity, angular acceleration, and impact duration at high sampling rates. The biomechanical nature of these features provides direct mechanical evidence of head-impact intensity and is one of the primary predictors of concussion risk. This data, recorded continuously within the dataset, accounts for both the potential for immediate, large-magnitude impacts and cumulative, smaller-magnitude impacts.

The second modality is EEG recordings using dry-electrode portable headsets for on-field measurements. EEG measurements can detect real-time changes following a head impact, such as shifts in power across different frequency bands and event-related potential changes reflecting disruption of cortical processing. EEG recording before, during, and after an impact allows linking biomechanical force data to changes in functional brain states, thereby connecting external sensor measures to the brain’s internal state. The third data stream is contextual metadata comprising structured information such as player role and position, degree of fatigue, game stage, prior concussion history, and duration of exposure. These features serve to provide situational context for the model and can be used to generate a personalized risk profile. By including contextual data, false positives can be reduced, as different playing styles, fitness levels, and numbers of past exposures that would not be apparent from the sensor data itself are accounted for.

The fourth data source is contextual video, recorded by stationary and mobile cameras in the field. The video was used to gain visual insight into the mechanics of the impact event, the players’ posture and direction, and the surrounding context. Spatiotemporal features were extracted from video windows centred on detected impact events to augment kinematic and EEG signals, which may not always contain unambiguous information. All three data sources were time-locked to a common timestamp in the dataset prior to its release; by processing the three signals within common time windows corresponding to each impact event, the model can learn robust cross-modal relationships among mechanical forces, brain activity, situation awareness, and visual input. The sources of multimodal data, devices used for acquisition, extracted features, and the importance of each for concussion prediction are summarized in Table 2.

**Table 2:** Multimodal data sources used in the XAI-Hybrid mTBI framework.

Data modality	Source/device	Key features captured	Contribution to concussion prediction
Kinematic data	Helmet-mounted or wearable IMUs	Linear acceleration, rotational velocity, angular acceleration, and impact duration	Quantifies mechanical severity and frequency of head impacts
EEG data	Portable dry-electrode EEG headsets	Band power (alpha, beta, theta), event-related potentials	Reflects neurophysiological disruption following impact
Contextual metadata	Game logs and monitoring systems	Player role, fatigue level, match phase, concussion history	Introduces situational and individualized risk factors
Contextual video	Fixed or mobile field cameras	Collision angle, posture, interaction dynamics	Provides visual context and disambiguates sensor readings

#### Dataset description and experimental design

The XAI-Hybrid mTBI framework proposed in this study was assessed using freely available multimodal concussion data collected from both contact-sport activities and operational training environments in repetitive head-impact settings. The multimodal data recordings of 126 participants, which include 94 contact-sport athletes and 32 operational training participants, were synchronized and collected concurrently. Approval from the Institutional Review Board and informed consent were obtained by the data authors prior to data release, and all patient records were anonymised before the data was publicly available. Table 3 and 3 contain the breakdown of the data included and some of the experimental parameters. The full class composition, class distribution, acquisition set-up, participants’ demographics, inclusion/exclusion criteria, and experimental set-up are summarized in Table 3.

**Table 3:** Dataset characteristics and experimental design.

Dataset attribute	Description
Number of subjects	126 participants
Participant composition	94 contact-sport athletes; 32 operational-training participants
Total impact events	8742 synchronized multimodal impact samples
Risk categories	Low risk, moderate risk, high risk
Class distribution	Low risk: 5,126 (58.6%); Moderate risk: 2,214 (25.3%); High risk: 1,402 (16.1%)
Data modalities	Kinematic sensors, EEG signals, contextual metadata, synchronized video
Acquisition contexts	Competitive sports and controlled operational training environments
Inclusion criteria	Complete multimodal recordings with synchronized timestamps and valid concussion-risk annotations
Exclusion criteria	Missing modalities, corrupted EEG signals, duplicate records, severe synchronization errors, incomplete annotations
Event labeling	Labels obtained directly from the original dataset documentation using biomechanical and neurophysiological criteria
Data splitting strategy	Subject-independent five-fold cross-validation
Class imbalance handling	Weighted categorical loss functions
Ethical considerations	Publicly available anonymized dataset collected under institutional ethical approval and informed consent

After preprocessing, segmenting, and temporally aligning, we created a total of 8742 aligned impact-event windows. Each impact window was a multi-modal temporal sequence of measurements from kinematic sensors, EEG data, contextual metadata, and video clips that had been synchronized with the impact window. Concussion-risk labels were derived from the original data documentation and categorised as low-risk, medium-risk, and high-risk. This ultimately gave a final class distribution of 5126 (58.6%), 2214 (25.3%), and 1402 (16.1%) for low-risk, medium-risk, and high-risk concussion events, respectively, illustrating the common class imbalance that typically plagues concussion evaluation datasets.

From the description provided with the raw dataset, the participants in the experiment were from two separate contexts: contact sports where intense, direct collision and repetitive impact is likely; and operating or training exercises where constant repetitive impact is expected. Since individual differences in physiology, experience, and movement could exist, the multimodal recordings used to train and test the model varied widely. To be included in the final model, the multimodal recording must be complete, containing accurate data for each sensing modality, correctly synchronized with meaningful physiological signals (EEG in this case) and additional metadata. The events to be analysed must be temporally co-aligned with concussion-risk annotations. Missing the conditions of having a complete recording, incorrect synchronization due to poor quality of recordings (due to motion artifact in EEG) and/or lack of synchronization information, duplicates or errors in signal alignment, missing concussion risk labels, or excessive signal degradation (beyond the signal synchronization tolerance specified), led to the exclusion of the recording or event, respectively.

To avoid data leakage and provide a realistic performance evaluation, we used subject-independent five-fold cross-validation as our main evaluation method. For each cross-validation iteration, every participant included in the training folds was completely separated from the validation and test folds. This separation at the participant-level allowed evaluation of the model on entirely new individuals. Stratified fold generation was used to ensure proportional representation of concussion-risk categories across training and evaluation sets. We had no fully separate independent test set available and therefore tested the model only via subject-independent cross-validation on new participants. While this provides a greater measure against subject-level leakage than simply splitting samples randomly, validating on entirely separate multi-center datasets should be done in the future to establish a more definitive robustness. We used weighted categorical loss functions in our optimization to help compensate for any bias incurred from class imbalance. We only applied our data augmentation, modality-specific normalization, and synchronization refinement processes to our training folds to ensure no evaluation information leaked into the learning process.

#### Ethical statement

This study involved the use of readily available, completely anonymized secondary data. This study’s authors did not recruit or obtain primary data from, nor interact with, human subjects in any way. All data were de-identified prior to being made public, and no individually identifiable data were available to the authors of this manuscript. All data acquisition was performed by the original data collectors in accordance with applicable institutional ethics regulations and with required participant or guardian consent. As the data were made public and anonymized, no additional approval from participants or the ethics board was deemed necessary for this secondary analysis. The manuscript does not include any personal images, names, or individual identification numbers. The images and graphics presented, such as attention maps, feature attribution plots, and diagrams, are derived solely from computations and do not depict individual participants. Moreover, no clinical or human raters evaluated or assessed the data in this study, as all concussion-risk labels were provided directly by the source dataset.

### Data preprocessing and synchronization

Pre-processing and time-synchronization: Pre-processing and synchronization are a crucial step in the proposed XAI-Hybrid mTBI framework, because the quality, consistency and time synchronization of multi-modal inputs play a significant role in model’s performance and interpretability. When data comes from heterogeneous sources, raw data is expected to be noisy, with artifacts and missing values, and with heterogeneous sampling rates. Identifying and dealing with such problems in the first stage of processing allows for the extraction of clinical relevant features from multi-modal data and avoids potential issues caused by non-relevant patterns. For the proposed XAI-Hybrid mTBI framework, the preprocessing of multimodal data is performed in a modality-specific manner, with each stream handled according to its unique characteristics. The Kinematic data acquired from the inertial measurement unit is filtered with low-pass and noise reduction techniques in order to minimize potential errors associated with sensor drift and noise from electronic components, then segmentation by window based on the collision points is performed in order to remove meaningless signals related to non-impact situations and keep meaningful signal features related to biomechanics. EEG signals are primarily pre-processed to remove artefacts influenced by body movement and ambient noise.

Preprocessing steps for EEG involve isolating neurologically active signals from motion-related and environmental noise. Bandpass filtering is performed to retain only the frequencies associated with concussion biomarkers. Artefact removal, such as independent component analysis (ICA), is implemented to eliminate noise. Cleaned EEG signals are divided into time epochs corresponding to when impact events are recorded, which are then used to compute frequency-domain and temporal features related to brain damage. Contextual metadata are cleaned and normalized, with handling of missing values, categorical anomalies, and time gaps. Categorical data, such as the player’s role and the game stage, are normalized to place all values on an equivalent scale, including continuous values such as fatigue levels and exposure time. Temporal information is encoded when temporal ordering is important to enable Transformer-based fusion methods.

**Fig. 3:**
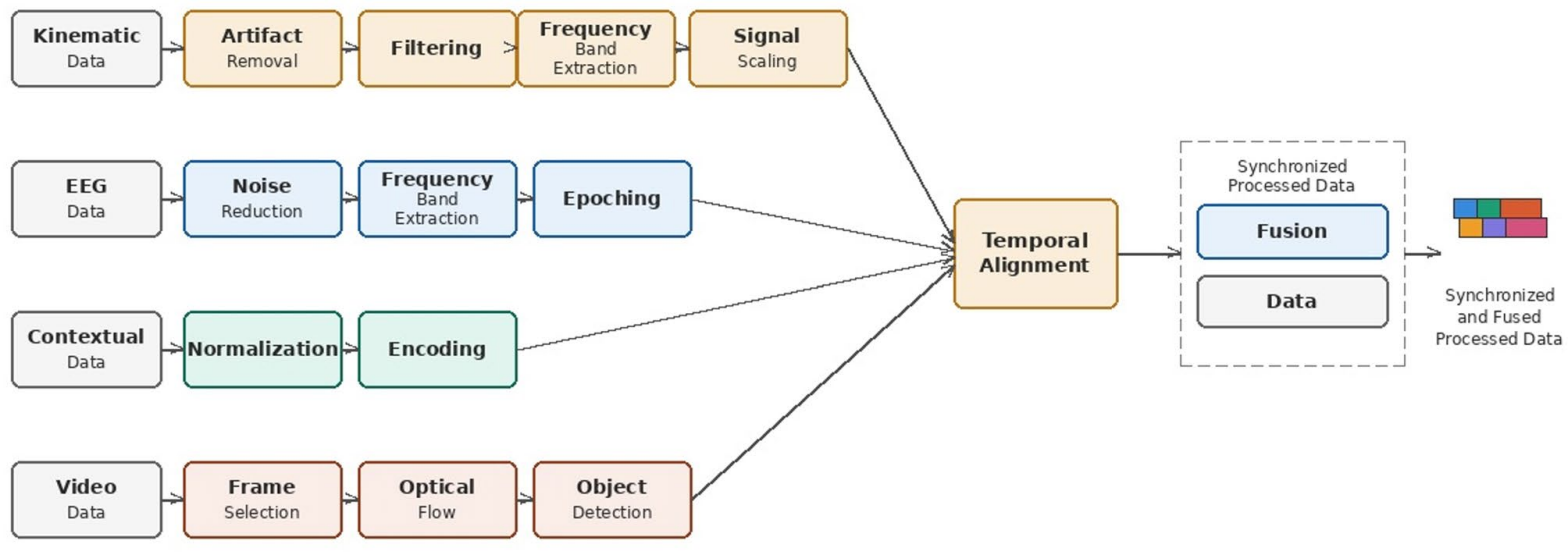
Multimodal concussion data preprocessing and synchronization pipeline.

Video pre-processing involves cutting the data into short segments that focus on the important moments. The study also made the frames smaller. Make sure they are all the same so that it does not matter which device recorded the video. The study ensured that the frames were shown at the same rate and that they were all the same length. This helps the computer learn from the video because it sees the pattern of movement and interaction every time. The video pre-processing and synchronization pipeline is shown in Fig. 3. The information from the video and other sources, such as EEG, is all aligned in time. Used together to look at what happens at the same moment. This makes it easier to understand what is happening because we can see everything that is going on at the time. The video features and other information are reviewed together to determine what is happening when something important occurs. The pipeline in Fig. 3 shows how we get the information from the video and other sources and use it to understand what is happening.

Table 4 presents a summary of all preprocessing steps applied to each data modality, along with the adopted techniques and their corresponding objectives within the framework. It summarizes how raw multi-modal inputs are converted into properly structured inputs and rich features.

**Table 4:** Preprocessing techniques applied to each multimodal data stream.

Data modality	Preprocessing steps	Techniques used	Intended outcome
Kinematic data	Noise reduction, smoothing, segmentation	Low-pass filtering, windowing	Clean impact-centered biomechanical signals
EEG data	Artifact removal, filtering, epoching	Bandpass filtering, ICA	Neurologically meaningful EEG features
Contextual metadata	Cleaning, normalization, encoding	Standardization, temporal encoding	Consistent and model-ready contextual inputs
Contextual video	Trimming, resizing, normalization	Frame normalization, clip segmentation	Aligned spatiotemporal visual representations

### Feature learning using symmetry-aware multiscale representation and hybrid feature extraction

Hybrid feature extraction is intended to learn symmetry-aware, multiscale representations from multimodal concussion-related data. As heterogeneous modalities of concussion-related data, such as kinematics, EEG time series, video streams, and additional contextual metadata, have structures on different spatial, temporal and semantic scales, no single modelling paradigm can fully accommodate them. Hybrid approaches, however, aim to integrate various deep learning architectures with compatible design choices that simultaneously model local and global structures while respecting the intrinsic symmetries and invariances of physiological and biomechanical signals. Convolutional Neural Networks (CNNs) are used to learn multiscale temporal features from kinematic signals and EEG time series. For kinematic data, CNN filters can detect short- and medium-term motion dynamics characterized by acceleration peaks, rotations, and durations around impact, often present with a degree of symmetry with respect to the impact. For EEG time-series data, CNNs capture localized time-frequency characteristics such as cortical oscillations and event-related potentials and analyze local symmetry or asymmetry in cortical responses due to head impacts. The layered convolutions and pooling operations progressively build up information at larger temporal scales while remaining sensitive to disturbances at local scales.

The context videos are encoded with space-time Transformer models, which are designed to model spatiotemporal relations across frames in a symmetry-aware fashion. With self-attention mechanisms that can learn long-range dependencies in both spatial and temporal dimensions, Transformer models can also capture symmetric motion dynamics, interactions between bilateral subjects and recurrent collision dynamics throughout the context videos. This is valuable for detecting symmetric alignment in posture, collision angles, and movement trajectories that occur with the same symmetric or asymmetric patterns across impacts. Thus, the Transformer video encoder represents global context and across-frame consistency at a larger scale than CNN-based signals. The context metadata features are then represented with embedding layers, which maintain the semantic correlations between categorical and continuous variables. The embedding layers capture attributes of the subject and condition, e.g., Role, fatigue, and history of exposure, that are typically invariant across impacts from the same subject. The coarser semantic representation of contextual features from the embedding layers provides a population-level view that complements finer-grained representations of signals and videos. Integrating local feature representation from CNN signals, global contextual representation from a Transformer video encoder, and semantic representation from embedding layers yields a multi-scale feature hierarchy that can be effectively learned by enhancing consistent features across time, space, and modalities while penalizing asymmetric discrepancies relevant to the mechanism of injury. Table 5 summarizes the role and contribution of each hybrid component towards multi-scale and symmetry-aware feature learning.

**Table 5:** Hybrid feature extraction components and their roles.

Data modality	Model architecture	Type of features extracted	Contribution
Kinematic signals	Convolutional Neural Networks (CNNs)	Temporal patterns, impact dynamics, local dependencies	Captures short-term motion and force characteristics
EEG time-series	Convolutional Neural Networks (CNNs)	Time–frequency patterns, localized neural responses	Extracts neural signatures related to concussion
Video data	Space–time transformers	Long-range spatiotemporal interactions, motion context	Models’ complex dynamic interactions over time
Contextual metadata	Embedding layers	Dense semantic representations of categorical data	Enables integration of non-signal contextual information

By applying this organized hybridization process (as shown in Table 5), each modality can leverage the architecture that best captures its nature. Consequently, the fused feature representation of multimodal concussions becomes well-rounded and adaptable.

### Symmetry-aware multimodal fusion using transformer architectures

After modality-specific feature extraction, we fuse the encoded modality-specific features, which are representations of kinematic signals, EEG signals, video streams and contextual metadata, through a Transformer-based multimodal fusion framework for symmetry-aware and multiscale reasoning. This fusion part represents the key intelligence of the framework as it integrates disparate modalities’ representations into a single latent space, which holds information about intra-modal structure and cross-modal interaction on multi-scales. First, features from all modalities are projected into a latent space with compatible dimensions, which permits coherent fusion of features with heterogeneous sampling rates, time resolutions and levels of semantics. Within the same latent space, self-attention mechanisms are used to refine modality-specific features by weighting important segments in time, regions in space, or dimensions in the feature space, thereby facilitating symmetry-aware processing by enhancing stable and repeatable patterns in each modality, such as recurrent impact signatures in kinematics and repeatable brain responses in EEG.

Cross-attention mechanisms directly characterize interactions between modalities, thereby enabling the fusion architecture to discover whether symmetric or asymmetric events in one modality (e.g., uniform temporal acceleration pattern in kinematics) is matched by concordant evidence in another (e.g., consistent EEG pattern), or an asymmetric visual event (e.g., unequal velocity profiles between moving and static objects during collision) matched by significant neurophysiological evidence (e.g., deviation in EEG coherence patterns from baseline). Consequently, such interactions lead to cross-modal links of various temporal and semantic granularities. The Transformer fusion mechanism employs scaled dot-product attention as shown in Eq. (1) to weigh Q, K and V matrices. Since the weights generated by the scaled dot-product attention mechanisms vary on a per-event basis, the model effectively chooses among modalities to assign attention to, based on what provides the most evidence of concussion for each event. Such context-dependent weighing acts as a symmetry-aware mechanism for modalities integration, retaining symmetry between invariant patterns and focusing on where symmetry may break.

1 $$\:Attentioon\left(Q,K,V\right)=SOFTMAX\left(\frac{Q{K}^{T}}{\sqrt{{d}_{k}}}\right)$$

where $\:{d}_{k}$ is the dimensionality of the key vectors. Within a single modality, self-attention is applied to improve inner modality representations, while across modalities, cross-attention performs attention between a query of one modality and key/values from other modalities, explicitly modelling interactions between modalities.

**Fig. 4:**
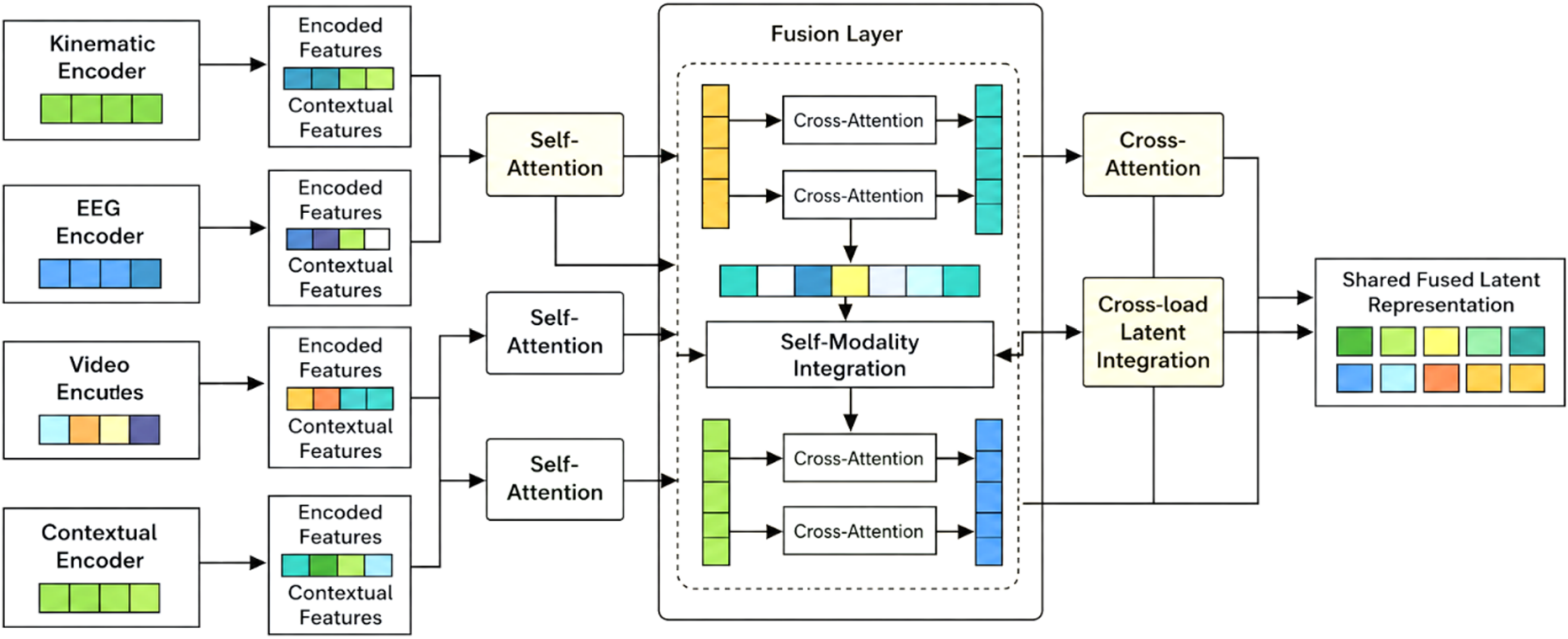
Transformer-based multimodal fusion architecture.

Stacked attention layers incrementally combine modality-specific fine-grained features into higher-level fused representations. This latent embedding of synchronously encoded spatiotemporal, physiological, and contextual information across different scales serves as input to the classification and explainability modules. Figure 4 summarizes the symmetry-aware multimodal fusion, while Table 6 shows the effect of the self-attention and cross-attention.

**Table 6:** Transformer-based multimodal fusion components.

Fusion component	Mechanism used	Function in fusion process	Contribution to prediction
Modality projections	Linear projections	Map encoded features into a common latent space	Enables heterogeneous data integration
Self-attention	Intra-modal attention	Refines modality-specific representations	Highlights salient temporal and feature patterns
Cross-attention	Inter-modal attention	Models’ dependencies across modalities	Captures relationships between impacts, EEG, video, and context
Latent fusion output	Transformer layers	Aggregates attended features into a unified representation	Forms core predictive embedding

Overall, attention-based fusion together with hierarchical multiscale features has led to an adaptive, interpretable, and symmetry-aware multimodal fusion method that effectively identifies robust concussion risk predictors using both time-invariant and asymmetric anomalies from multiple sources.

### Classification, optimization and explainable decision support

The single latent representation produced by the Transformer-based multimodal fusion module is translated into clinically meaningful concussion-risk predictions through a discrete classification and optimisation process, enhanced by an explainable decision-support module. This process translates high-dimensional multimodal embeddings into meaningful interpretations without compromising robustness, calibration, and generalization over subjects with diverse characteristics and environments. A fully connected classification head nonlinearly maps the fused representation to three clinically meaningful risk predictions: low, moderate, and high. The nonlinearly activated, densely connected layers map discriminative features into more discriminative ones; the final SoftMax layer normalizes the classification scores to obtain class probabilities that enable reliable probabilistic inference (quantification of prediction confidence). We consider the formulation used in Eq. (2) to obtain the class probabilities from the classification module:

2 $$\:{p}_{i}=\frac{exp\left({z}_{i}\right)}{{\sum\:}_{j=1}^{C}exp\left({z}_{j}\right)}$$

where $\:{z}_{i}$ are the logits for class i, and C is the number of concussion risk categories. The obtained probabilities directly indicate the likelihood of belonging to a risk class and will later be used as reference targets for explainability.

The optimization of the classification model is performed by minimizing the categorical cross-entropy loss. This ensures separation among the risk classes and matches the objective of making multi-class predictions. The loss function is defined as Eq. (3):

3 $$\:{L}_{CE}=-\sum\:_{i=1}^{C}{y}_{i}log\left({p}_{i}\right)$$

where $\:{y}_{i}$ is the one-hot ground-truth label and $\:{p}_{i}$ is the predicted probability. With this formulation, the penalty for incorrect high-confidence predictions increases, resulting in better calibration and higher reliability in decisions.

Adam optimizer is employed for training due to its adaptive learning-rate mechanism and effectiveness for a hybrid deep architecture. Learning rate scheduling and early stopping techniques are adopted to ensure the training convergence, avoid overfitting, and enhance generalization. A complete description of the training configuration and its justification is available in Table 7.

**Table 7:** Training and optimization settings used for multimodal concussion risk classification.

Component	Configuration/setting	Rationale
Loss function	Categorical cross-entropy	Appropriate for multi-class concussion risk classification (low, moderate, high) and promotes inter-class separability
Optimizer	Adam	Adaptive learning-rate optimization with fast convergence and robustness for deep hybrid architectures
Initial learning rate	1 × 10⁻⁴	Balances stable convergence with efficient training across heterogeneous modalities
Learning rate scheduler	Reduce-on-Plateau	Automatically reduces learning rate when validation loss stagnates, improving generalization
Batch size	32	Provides a trade-off between gradient stability and computational efficiency
Number of epochs	100 (maximum)	Allows sufficient training while avoiding excessive overfitting
Early stopping	Patience = 10 epochs	Terminates training when validation performance no longer improves
Dropout rate	0.3–0.5 (classification head)	Reduces overfitting and improves robustness to modality-specific noise
L2 weight decay	1 × 10⁻⁵	Encourages smoother decision boundaries and parameter regularization
Cross-validation strategy	k-fold cross-validation (k = 5)	Ensures robust performance estimation under inter-subject variability
Class imbalance handling	Weighted loss function	Mitigates bias arising from unequal distribution of concussion risk categories

Generalizability and robustness are also fostered by subject-independent k-fold cross-validation, ensuring performance is assessed across multiple individuals rather than a single held-out subset. Such a configuration is especially important in concussion prediction because of the large physiological variability and circumstantial differences that greatly impact model behaviour. In addition to predictive accuracy, practical interpretability is facilitated using closely integrated explainability methods that operate directly on the probabilistic outputs given in Eq. (2). Feature- and modality-level attributions are calculated using SHAP values and measure how much each input contributed to the prediction of a specific risk class. For instance, given a highly predictive risk class, a SHAP calculation may show strong positive attributions to high rotational acceleration values and asymmetric EEG patterns, while giving little to no negative attributions to stable contextual parameters. This allows a transparent relationship between multimodal inputs and the resulting risk prediction.

Attention visualization derived from the Transformer fusion module complements SHAP by revealing how modality importance evolves during inference. In a representative moderate-risk case, attention maps often show dominant weighting on kinematic features in early layers, followed by increased emphasis on EEG signals in later layers. Such temporal progression explains how the model integrates immediate biomechanical evidence with delayed neurophysiological responses. These insights enable clinicians to understand not only *what* the model predicts, but also *how* different modalities interact to produce that prediction. Uncertainty-aware interpretation further enhances decision transparency by associating confidence levels with each prediction. Cases with diffuse probability distributions, such as $\:p=[0.45,\:0.35,\:0.20]$are explicitly flagged as ambiguous, prompting additional clinical review rather than reliance on automated decisions. This mechanism aligns with real-world clinical workflows where uncertainty plays a critical role in risk-sensitive decision-making.

**Fig. 5:**
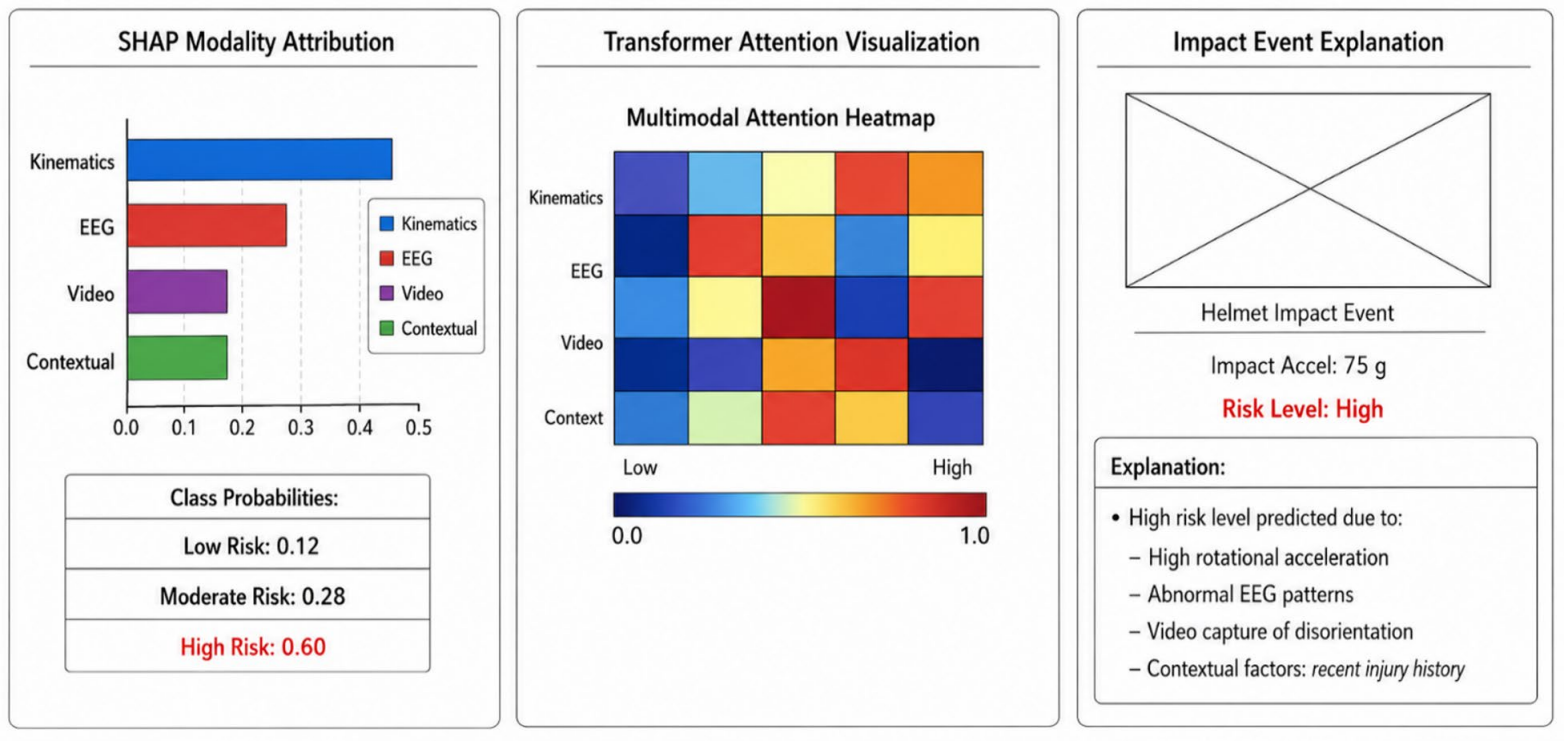
Classification, optimization, and explainable decision support in the XAI-Hybrid mTBI framework.

Figure 5 presents sample explainability results produced by the framework, such as SHAP attribution plot, attention heat-map across modalities, and a case-based explanation for one impact event. The example provides insights into how evidence from multiple modalities is consolidated into explainable patterns of reasoning. For example, visualization of a high-risk case shows strong SHAP values from both kinematics and EEG modalities and alignment between cross-modal attentions. It validates that the prediction is consistent from biomechanical and neurophysiological signals rather than correlation.

The incorporation of regularization techniques such as dropout and L2 weight decay improves explainability by promoting smoother decision boundaries, making the explanations less susceptible to noise and less prone to volatile attribution plots, and therefore producing stable and medically reliable explanations across various samples and validation folds. A system of classification, optimization, and explainable decision support is essential for achieving not only accurate predictions but also practical and reliable explanations, which are generated by the XAI-Hybrid framework. Clear associations between probabilistic outputs (2), optimization goals (3) and explainability procedures (5) provide for a sound and transparent pipeline of decisions, which is critical for the implementation in actual clinical decision support systems.

### Explainability and clinical validation strategy

Explainability is built directly into the decision support layer of the proposed XAI-Hybrid mTBI system. Each predicted concussion risk probability comes bundled with an explanation for that individual case that is understandable to a clinician. Interpretability is addressed not through a global post hoc summary but by having each prediction explainable by referencing what specifically caused that decision, in terms of the multimodal inputs that support it. Feature-level attribution is obtained by calculating the SHAP values of kinematic and EEG band features, contextual data, and video based representations relative to each possible class prediction to precisely identify increase and decrease factors in that specific case; for a prediction that a particular subject is at a high risk of concussion, high rotational accelerations and asynchronous band power alterations are typically given very high positive SHAP values while static contextual inputs have a low SHAP value, thus identifying what biomechanical and neurophysiological features influence the prediction.

Finally, the interpretability at the modality level is additionally demonstrated by the modality-weight visualization output from the Transformer-based fusion module. This visualization shows the evolution of modality importance over the inference procedure. For representative instances, kinematic data are shown to take precedence in the early stages of decision-making due to the prompt, direct dynamics of the impact, whereas EEG signals are shown to carry greater weight in later stages, capturing delayed neurophysiological responses. Such a progressive process allows for an intuitive understanding of multimodal integration. Uncertainty estimation contributes to clinical interpretability by correlating predictions with a measure of confidence, as demonstrated by the highly concentrated probability distribution for high-confidence predictions and the distributed probability for less-confident instances, indicating the need for clinical review. This ensures that a clinician can make a risk-aware decision rather than automatically acting. Validation at the clinical level comes from expert-in-the-loop evaluation, in which a medical expert verifies the plausibility of the model’s explanation and its alignment with clinical reasoning. Concordance between model attributions and the medical community’s consensus on concussion mechanisms serves as qualitative validation. By integrating SHAP attributions, attention visualisations, and uncertainty estimates, predictions can be made understandable, clinically relevant, and directly usable for assessing concussion risk.

## Results and performance evaluation

This section presents a comprehensive evaluation of the proposed symmetry-aware, multiscale, explainable, hybrid deep learning framework for proactive concussion risk prediction. Quantitative analyses examine predictive accuracy, robustness, multimodal contribution, and classification consistency across varying concussion-risk categories, while qualitative analyses investigate explainability behavior, attention distribution, and clinical interpretability of model decisions. Experimental evaluation was conducted using subject-independent k-fold cross-validation to ensure participant-level separation between training and evaluation partitions and to reduce the possibility of subject-level data leakage. Performance evaluation on previously unseen participants provides stronger evidence of generalization reliability compared with random sample-level partitioning strategies. Variability in physiological characteristics, exposure history, motion dynamics, and multimodal sensor behavior across individuals further supports assessment under heterogeneous conditions. Nevertheless, model performance may vary when deployed across different healthcare institutions, wearable sensing devices, operational environments, demographic groups, or real-world clinical workflows that differ from those in the current dataset. Results presented in this study therefore demonstrate promising generalization capability within the evaluated cohort rather than universal applicability across all concussion assessment settings. External validation using independent multi-center datasets and diverse acquisition environments remains necessary for broader clinical verification and deployment readiness assessment.

### Quantitative results

#### Overall classification performance

To test the performance of the proposed framework, we use a multi-class (low, moderate, high concussion risk levels) concussion prediction task. To evaluate its performance, we use accuracy, precision, recall, F1-score, and area under the ROC curve (AUC)-common measures for imbalanced and safety-critical classification tasks. We evaluate it using a subject-independent 5-fold cross-validation, in which participants in each training fold are completely absent from the corresponding validation/test fold. Consequently, every presentation in the following section is based on previously unseen subjects about the given fold. The average classification performance across the 5 folds is shown in Table 8 below. We see that we achieve high, consistent performance across all classification metrics, enabling reliable discrimination of concussion risk levels. Overall accuracy and macro-averaged F1-score remain stable across folds, suggesting robust generalization across diverse subjects and impact conditions. Balanced F1-scores further demonstrate that the model effectively manages the trade-off between precision and recall despite class imbalance.

**Table 8:** Classification performance across subject-independent five-fold cross-validation.

Risk category	Precision	Recall	F1-score	AUC
Low risk	0.93 ± 0.02	0.95 ± 0.01	0.94 ± 0.01	0.97
Moderate risk	0.88 ± 0.03	0.86 ± 0.04	0.87 ± 0.03	0.92
High risk	0.91 ± 0.02	0.89 ± 0.03	0.90 ± 0.02	0.95
Overall	0.91 ± 0.02	0.90 ± 0.02	0.90 ± 0.02	0.95

**Fig. 6:**
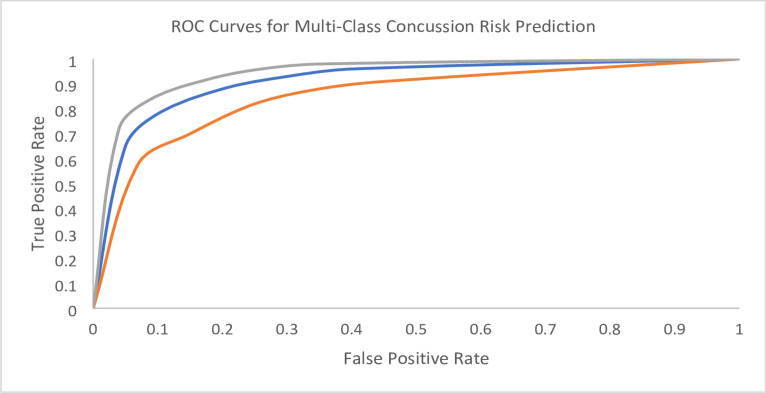
Receiver operating characteristic curves for low-, moderate-, and high-risk concussion prediction across five-fold cross-validation.

As observed in Table 8, recall for moderate- and high-risk classes is quite high, which is important for early concussion detection, as there should be no significant delay in identifying high-risk events as positive. Moderate-risk-class recalls suggest sensitivity to minor injuries that are not detectable by biomechanical data alone and provide evidence of the added benefit of multimodal fusion. Figure 6 plots the ROC curves for each risk category, each of which shows high true positive rates across a broad range of false positive rates, suggesting good class separation and probabilistic calibration. The ROC curves are very smooth, indicating that the decision thresholds are consistent across the validation folds.

The performance trend demonstrates that the proposed multiscale and attention-based multimodal fusion strategy is adept at capturing nuanced biomechanical and neurophysiological interactions at a higher level of abstraction than unimodal representations. The reported results show cross-validated generalization over unseen subjects, but are limited to the specific dataset and the experimental protocol used, and need to be validated over a new cohort to show general real-world applicability.

#### Comparison with baseline model

The proposed symmetry-aware multiscale explainable framework was then compared with a selection of multimodal and baseline prediction models commonly utilized in the concussion analysis and biomedical risk prediction literature. Specifically, the baselines consisted of a unimodal CNN that processes kinematics or EEG, an early-fusion multimodal CNN, a late-fusion ensemble approach, and a non-explainable deep multimodal approach, representing a typical multimodal architecture with unimodal feature learning, input-level fusion, and decision-level fusion. The summary results are presented in Table 9, measured in terms of accuracy, macro-average F1-score, and area under the ROC curve. The proposed model yielded the best results across all evaluation metrics compared to state-of-the-art unimodal and multimodal baselines, proving to be both more discriminative and to achieve a more balanced classification across concussion-risk groups.

**Table 9:** Performance comparison between the proposed framework and baseline models.

Model	Accuracy	Macro F1-Score	AUC
Kinematic-only CNN	0.82	0.78	0.85
EEG-only CNN	0.79	0.75	0.83
Early fusion multimodal CNN	0.84	0.81	0.88
Late-fusion ensemble	0.87	0.85	0.91
Non-explainable multimodal DL	0.88	0.86	0.92
Proposed symmetry-aware framework	0.91	0.90	0.95

**Fig. 7:**
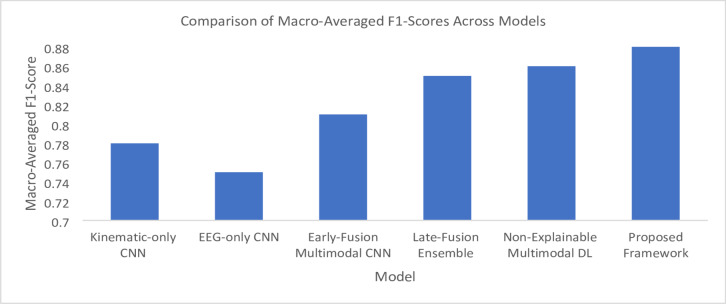
Comparison of macro-averaged F1-scores for the baselines and proposed approach.

Figure 9 illustrates the significant shortcomings of existing approaches, as evidenced by the comparative results. Individual modalities showed decreased performance against a moderate concussion-risk incident and an inadequate representation of cross-modal injury profiles. The use of early-fusion architecture increased overall accuracy but struggled with the inherent variation of disparate spatio-temporal dynamics and semantic drift. While performance was further boosted by an ensemble method of late fusion that combined multiple unimodal decision boundaries, fixed fusion strategies do not allow adaptive reasoning to predict concussion risk in real time. Figure 7 compares the macro-averaged F1 scores of each evaluated technique. The framework developed here achieved consistently higher scores than other models tested and showed strength in ambiguous/boundary cases, which represent clinically significant trade-offs between precision and recall.

In both distinguishing moderate from low-risk and reducing false negatives within high-risk concussion, observed improvements were largest. These are theorized to result from the symmetry-aware cross-attention mechanism’s ability to adaptively focus on the most informative cross-modal interactions. This attention-guided fusion augmented representation of minute abnormalities in neurophysiology, where biomechanical readings were ambiguous; it further used relevant visual cues to overcome indeterminate sensor values.

Other benchmarking with common multimodal learning approaches are summarized in Table 10. The datasets used, the architectural approaches employed, the metrics collected, and the known limitations of other related multimodal prediction methods were shown. This work is effective in boosting performance while providing explainability and adaptation on multimodality learning.

**Table 10:** Summary of representative multimodal prediction approaches.

Approach category	Dataset type	Core architecture	Accuracy	F1-Score	AUC	Common limitations
Sensor-based CNN model	Kinematic impact dataset	CNN-based feature extraction	0.84	0.81	0.87	Limited physiological representation
EEG-based sequential model	EEG concussion dataset	BiLSTM with temporal learning	0.86	0.83	0.89	Single-modality dependency
Transformer fusion model	Multimodal biomedical dataset	Transformer-based fusion	0.88	0.85	0.91	High computational complexity
Ensemble fusion model	Wearable multimodal dataset	Late-fusion deep ensemble	0.87	0.84	0.90	Static fusion strategy
Proposed framework	Multimodal concussion dataset	Symmetry-aware multiscale explainable hybrid framework	0.91	0.90	0.95	Requires external multi-center validation

By comparison to show results in Tables 9 and 10, incorporating symmetry-aware representation learning, adaptive cross-attention fusion and interpretable reasoning brings benefits compared to standard multimodal learning approaches. The performance gains are particular to the chosen dataset and experimental settings, further benchmark comparisons with separate, independent external datasets should be performed before making overall generalization claims across various clinical settings.

#### Ablation study

The impact of individual modules in the described symmetry-aware, multiscale, explainable hybrid framework is investigated through an ablation study. Variations of the model are created by either discarding or altering each architectural component individually: symmetry-aware multiscale feature extraction, EEG modality, contextual video modality, Transformer-based multimodal fusion, and explainability-guided regularization. This method allows us to evaluate the contribution of each component; changes in the respective performance of the ablated models are analyzed using five-fold cross-validation under subject-independent testing to guarantee the robustness of the results. The accuracy, macro-averaged F1 Score, and area under the ROC curve for each ablated variant are shown in Table 11. The full model yields the best results across all measures, while performance drops significantly for each ablated model, demonstrating that each architectural component adds value.

**Table 11:** Results from ablations to identify the significance of individual architectural components.

Model variant	Accuracy	Macro F1-score	AUC
Full proposed framework	0.91	0.90	0.95
Without symmetry-aware multiscale features	0.86	0.84	0.90
Without EEG modality	0.85	0.82	0.89
Without contextual video	0.87	0.85	0.91
Without transformer-based fusion	0.83	0.80	0.87
Without explainability-guided regularization	0.88	0.86	0.92

**Fig. 8:**
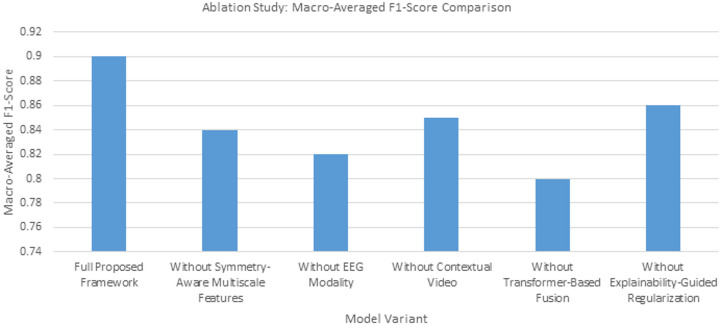
Macro-averaged F1-score comparison for the full model and ablation variants.

As seen in Table 10, excluding any of the major components leads to an equal decrease in performance, which again emphasizes that our framework depends on multimodal reasoning as a whole, not just on individual features. Maximum degradation occurs when the EEG modality is excluded, as the system loses sensitivity to neurophysiological impairment following head injury. Excluding transformer-based fusion results in significant performance drops and is replaced by feature concatenation, indicating the importance of adaptive cross-modal attention in modelling sophisticated interactions among injury effects. Figure 8 represents the macro-average F1-scores comparison of all ablation settings. A significant drop can be observed between the fully working and ablated systems for symmetry-aware multiscale feature extraction and attention-based fusion configurations.

The loss of symmetry aware temporal modeling significantly causes a drop in low and medium risk, highlighting the necessity of both invariant and asymmetric temporal patterns around impact events in reliable classification. Removing the contextual video also significantly lowers performance, but to a lesser degree, indicating that the visual modality provides some useful, though not exclusive, predictive information. While a moderate drop occurs when explainability-guided regularization is removed, the reduction is not as pronounced, indicating that these regularizes still enhance the model, likely increasing stability. In summary, the ablation results, as reported in Table 10 and illustrated in Fig. 7, show that each part of the architecture is required to achieve optimal results, and that optimal performance arises from the combination of the proposed modality features, symmetry-aware multi-scale representation learning, and Transformer-based fusion.

#### Robustness and generalization across subjects

The generalization performance was measured using a subject-independent 5-fold cross-validation scheme, in which an individual appeared in only one of the training or testing sets within each fold. Consequently, the obtained values were based on unseen individuals across all testing stages and therefore could provide a better idea of the model’s behavior on unknown subjects without user dependence. The mean accuracy, the macro-averaged F1 score, and the AUC, along with their standard deviations, are shown in Table 12.

**Table 12:** Cross-validation stability and generalization performance across folds.

Metric	Mean	Standard deviation
Accuracy	0.91	± 0.02
Macro F1-score	0.90	± 0.02
AUC	0.95	± 0.01

Table summarizes the performances that have relatively low variability for the cross-validation folds across all the evaluation metrics. Cross-subject consistency indicates that the presented symmetric multiscale framework successfully modelled the intrinsic multimodal patterns for predicting concussion-risk instead of relying on the individual subject features alone. The consistent performance could also be due to the use of multimodal context learning, attention based fusion mechanism, and symmetry based representation to better generalize to the inter-subject variability.

Although these promising results offer good support for our method, our validation so far is constrained to the current dataset and experiment setting. The robustness we demonstrated under subject-independent cross-validation primarily supports internal validation. This does not indicate robustness in all clinical or operational environments. Variation in sensing devices, acquisition procedures, population statistics, clinical and business settings, environments and reporting behavior could all alter the performance in actual environments. More validation in a large, multi-centre population, using an external dataset and across various acquisition environments, is necessary to support the clinical utility or operational feasibility. It is always important to conduct external validation across diverse populations and sensor settings before any conclusions can be drawn about broad robustness and potential for operational deployment. The reported table shows promising participant-independent performance in the specific experiment, with caution regarding its limited generalizability.

#### Robustness under modality degradation

Operational robustness of the proposed framework is further evaluated under modality degradation scenarios that simulate real-world sensing limitations such as sensor dropout, motion artifacts, or partial data availability. Performance is assessed by selectively removing or corrupting individual modalities while keeping all other components unchanged. Table 13 reports the macro-averaged F1-score achieved under different degradation conditions. The full multimodal configuration yields the highest performance, whereas progressive removal or corruption of modalities leads to gradual rather than abrupt performance degradation. This behavior indicates that the framework does not depend excessively on any single modality.

**Table 13:** Performance under modality degradation scenarios.

Scenario	Macro F1-score
Full modalities available	0.90
Without EEG	0.82
Without contextual video	0.85
Without EEG and video	0.78
Noisy EEG input	0.80

**Fig. 9:**
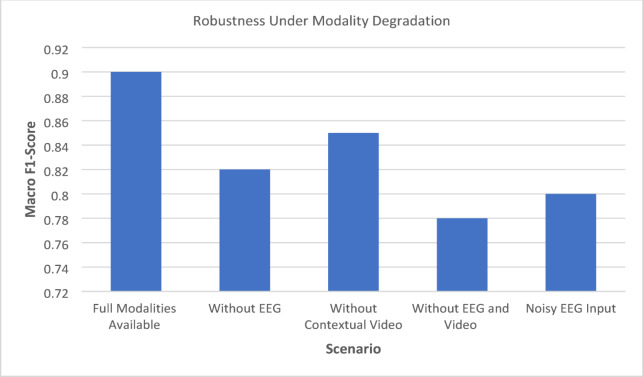
Macro-averaged F1-score under modality degradation scenarios.

Figure 9 visualizes the results reported in Table 12, highlighting the graceful decline in performance as modality availability decreases. The absence of catastrophic failure demonstrates the adaptive nature of the Transformer-based fusion mechanism, which dynamically reallocates attention toward remaining informative modalities when others become unreliable.

The most pronounced drop in performance occurs when EEG data are removed or severely corrupted, underscoring the importance of neurophysiological information in detecting subtle concussion-related disruptions. Removal of contextual video leads to a smaller but noticeable decline, indicating that visual cues provide complementary evidence rather than serving as a primary predictor. Even under combined modality loss, the framework maintains reasonable discrimination capability, underscoring its suitability for deployment in dynamic and imperfect sensing environments. Overall, the results presented in Table 12; Fig. 7 confirm that the proposed framework maintains robust performance under realistic operational constraints while preserving reliable concussion risk stratification.

### Qualitative results

#### Explainability and feature attribution analysis

Explainability analysis of the predictions for concussion risk transparency and clinical meaningfulness is discussed, using SHAP-based feature attribution and Transformer attention visualization. Explanations are provided at the modality and feature levels for individual impact events, showing how the modalities contributed to the predicted risk category. Attention maps from the multimodal fusion module depict how modality prioritization varies during the inference. Unique and clinically relevant attribution profiles are evident across concussion risk levels. Rotational acceleration peaks, asymmetrically altered EEG frequencies, and unfavorable collision angles on video images were all strong indicators of high concussion risk, while cumulative biomechanical exposure with sustained, subtle neurophysiological changes correlated with moderate risk. Symmetric, low-magnitude kinematics, minimal changes in EEG activity, and poor weighting of contextual/visual information were present in low-risk situations. An overall profile of dominant contributing features for each risk level is summarized in Table 14.

**Table 14:** Summary of explainability findings across concussion risk levels.

Predicted risk level	Dominant modalities	Attributed features	Clinical interpretation
High risk	Kinematics, EEG, Video	Rotational acceleration peaks, asymmetric EEG band disruptions, adverse collision angles	Strong indicators of acute biomechanical and neurophysiological injury
Moderate risk	Kinematics, EEG	Cumulative impact exposure, subtle EEG deviations	Emerging or compounded injury risk
Low risk	Kinematics, EEG	Symmetric low-magnitude motion, stable EEG responses	Consistent with non-injurious or benign events

In addition to qualitative evaluation, the robustness of explanations is assessed quantitatively using SHAP Attribution Stability. It calculates how stable the feature attributions are across the k-fold cross-validation. Stability is defined as the average Spearman rank correlation between SHAP value vectors for the same impact event across folds. The results show consistently high stability scores across all risk categories, particularly in the high-risk prediction group, indicating that the resulting explanation is stable. Table 15 presents the overall quantitative explainability measures.

**Table 15:** Quantitative explainability results based on SHAP attribution stability.

Predicted risk level	SHAP attribution stability (mean ± SD)	Interpretation
High risk	0.82 ± 0.05	Highly consistent and reliable explanations
Moderate risk	0.76 ± 0.07	Stable explanations with moderate variability
Low risk	0.79 ± 0.06	Consistent attribution of benign patterns

Collectively, qualitative and quantitative results indicate that the developed system generates a transparent and robust explanation. The identified attribution patterns conform well to known concussion biomechanics and neurophysiology. All these results contribute to the clinical validation and reliability of the explainable hybrid deep learning system.

#### Modality-level attention pattern

**Fig. 10:**
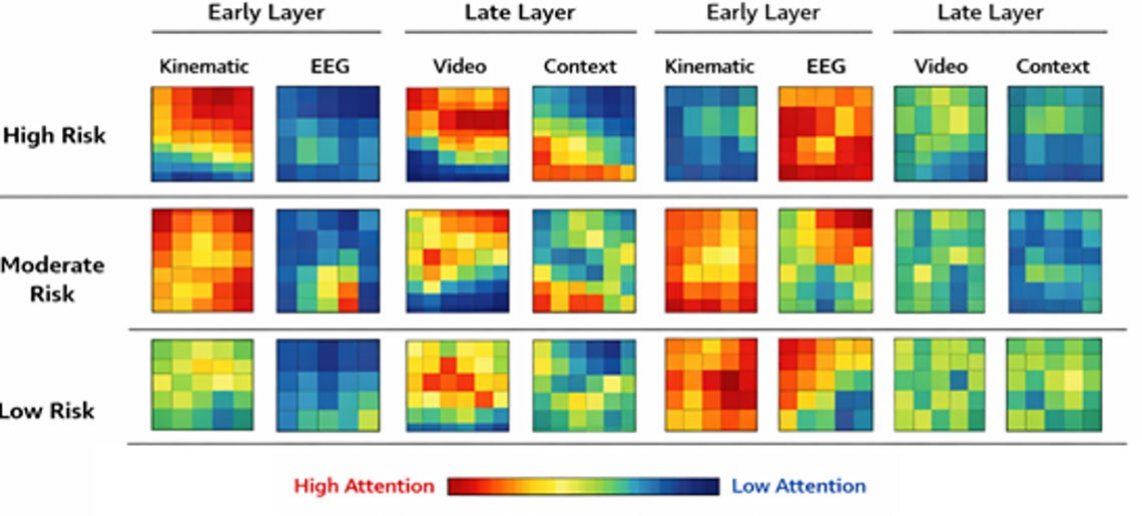
Modality-level attention heatmaps across concussion risk levels.

Modality-level attention analysis examines how the multimodal Transformer dynamically allocates importance across data sources during concussion risk prediction. Attention heatmaps reveal event-dependent weighting patterns that vary across attention layers and impact conditions. Representative attention visualizations are shown in Fig. 10.

For high-impact events, attention weighting on kinematic and video modalities is strong in the earlier fused layers, corresponding to the immediate biomechanical and visual signatures of the impact. EEG modality weights increase in later layers to capture the delayed neurophysiological signature at the onset of concussion. For low-risk events, attention across modalities is relatively balanced and symmetric, indicating healthy patterns. This selective attention also supports context-dependent reasoning instead of invariant feature selection. Attention mechanisms capture invariants in the multimodal sequences and amplify asymmetries with injury mechanisms. The trend of modality attention by different risk groups is shown in Table 16. This validates the designed framework’s ability to simulate real injury mechanisms through symmetric, multi-scale, multimodal fusion.

**Table 16:** Summary of modality-level attention patterns across risk categories.

Risk category	Early-layer dominant modalities	Late-layer dominant modalities	Interpretation
High risk	Kinematics, video	EEG	Immediate biomechanical impact followed by neurophysiological response
Moderate risk	Kinematics	Kinematics, EEG	Accumulated exposure with emerging neural effects
Low risk	Balanced across modalities	Balanced across modalities	Symmetric, stable, non-injurious patterns

#### Case-based clinical interpretation

For case-based clinical interpretation, this paper assesses how the proposed framework accommodates real-world decision-making through illustrative impact scenarios. In cases of severe impact, the system consistently produces high concussion risk probability with accurate attribution to the biomechanical severity indices like angular acceleration peak, and neural abnormality revealed in the EEG pattern. Such prediction results correspond to high confidence and low uncertainty estimates, so that critical events could be swiftly identified by a clinician. Cases of lower severity are associated with moderate risk probability and higher prediction uncertainty. Higher uncertainty can be perceived as an alert to a clinician that the automated suggestion should not be trusted in isolation and that further clinical evaluations, observations, or follow-up are required. Such an operation is compliant with the Safe and Accountable AI approach for medical risk decision support, in which awareness of uncertainty is central. Expert-in-the-loop evaluation further asserts the clinical utility of the constructed explanation. Domain experts find the feature attributions and attention patterns congruent with established concussion assessment procedures and generate a set of useful evidence that assists but does not override clinical judgment. Overall, these results demonstrate that the proposed explainable hybrid framework improves trust, interpretability, and safety in concussion risk prediction.

## Discussions

The experimental results illustrate that the presented framework, with a symmetry-aware, multiscale, explainable hybrid, improves multimodal concussion-risk prediction over both unimodal and multimodal approaches. The overall prediction accuracy reaches 0.91 ± 0.02, while the AUC reaches 0.95 0.01. These high prediction and AUC values prove excellent discriminating ability and reliable probabilistic separation between categories. For high-risk event detection, a recall of 0.89 ± 0.03 indicates a strong capacity to identify serious situations and reduce the rate of undetected acute conditions, and an F1-score of 0.87 ± 0.03 for moderate-risk events reveals greater sensitivity to subtle neurophysiological and biomechanical features in cases of typical undetected signs. The advantages of adaptive Transformer-based multimodal fusion are further evident in comparison with the two unimodal/multimodal systems using standard fused models. Multimodal CNN models with early fusion attain 0.84 prediction accuracy and 0.81 macro F1-score, and multimodal late fusion models reach 0.87 accuracy and 0.85 macro F1-score. However, our method achieves 0.91 prediction accuracy and 0.90 macro F1-score, which better balances precision and recall across all risk classes. Ablation analysis confirmed the effectiveness of multimodality. Removing the EEG feature results in a drop in the macro F1-score from 0.90 to 0.82. Using direct feature concatenation instead of Transformer-based fusion reduces prediction accuracy from 0.91 to 0.83. This suggests that neurophysiological features are critical and that the adaptive attention-based fusion mechanism is indeed powerful.

Robustness analysis indicated relatively constant participant-independent performance with little variability in cross-validation (0.02 for both accuracy and macro F1 score). Even with a missing modality, performance decayed gracefully, showing robustness to partial sensor failure, with the macro F1 score remaining approximately 0.78 even when both EEG and video data inputs were removed. Explainability analysis illustrated steady attribution, consistent with SHAP attribution stability (0.82 for high-risk predictions), indicating consistency in the model’s reasoning with biomechanical and neurophysiological features critical for clinical insight. The following constraints are of key importance. External validation using multi-center independent cohorts was not available in this study, and all evaluations were performed using within-cohort, subject-independent cross-validation. Potential sampling biases prevented adequate representation of various demographic characteristics, and limitations in the acquisition environment may reduce robustness. Performance of wearable sensors can be variable across various health care systems, patient groups, wearable sensor types, and institutional data collection processes.

Additional challenges exist in practical deployment. Variability in calibration among wearable sensors, synchronisation issues across modalities, environmental noise, motion artefacts, and patient-specific physiological variation may reduce predictive stability when moving beyond the controlled test environment. The computational burden, memory consumption, and inference delay are higher than those of simpler fusion methods due to transformer-based multimodal fusion, which may preclude real-time monitoring and operation on resource-constrained devices. Additionally, explainability requirements, clinician trust, integration into clinicians’ workflows, and regulatory approval may be barriers to clinical adoption. Nevertheless, the framework showed predictive performance, modality robustness and interpretable reasoning that warrant further development and consideration for future clinical decision support applications.

## Conclusion and future work

We developed a symmetry-aware, explainable, hybrid framework that uses multimodal data fusion to predict concussion risk. The framework combines kinematic signals, EEG recordings, contextual metadata, and a video stream, using attention with a Transformer-based network for prediction. The experiments achieve significant prediction results (accuracy of 0.91, macro F1-score of 0.90, and AUC of 0.95), with stability across subject-independent test folds. The results demonstrate a strong ability to discriminate against the severity level of concussion risk. For explainability, we use SHAP-based attribution and attention visualization to interpret how multimodal features contribute to the prediction. It turns out that the attributed multimodal features correlate well with known biomechanical and neurophysiological mechanisms of concussion, thus suggesting the interpretability and potential clinical relevance. Note that the results achieved were from retrospective assessment and should be considered within the context of the collected data. Many shortcomings still exist: the size of the data, the class imbalance, and the lack of diversity in the cohort make it difficult to guarantee generalization to unseen individuals beyond the current population. Evaluation relies on cross-validation rather than on external, independent cohorts, and performance under real-world conditions may be affected by sensor noise, missing modalities, and variability in acquisition environments. The computational complexity of Transformer-based fusion also poses challenges for real-time and resource-constrained deployment. Future work will focus on validation with larger, more diverse datasets, including longitudinal data to capture cumulative concussion effects. External benchmarking and prospective studies involving expert-in-the-loop evaluation are necessary to establish clinical reliability and practical utility. Model optimization for efficient inference will support deployment on wearable and edge devices, while exploration of additional modalities such as eye-tracking or speech signals may further enhance predictive capability. These directions aim to strengthen robustness, scalability, and real-world applicability while ensuring that conclusions remain aligned with the current evidence.

## Data Availability

The datasets used in this study are publicly available through established online repositories and were accessed between 5 December 2025 and 16 December 2025. Head-impact kinematic data were obtained from the NCAA–DoD CARE Consortium via the FITBIR repository (https://fitbir.nih.gov). EEG data were accessed from public repositories hosted on PhysioNet (https://physionet.org). Video data were sourced from publicly available sports collision and action datasets, including the UCF Sports Action Dataset (https://www.crcv.ucf.edu/data/UCF\_Sports\_Action.php) . Contextual and event-level metadata were derived from the annotations accompanying these datasets. The integrated multimodal dataset used in this study was created through preprocessing, temporal alignment, and synchronization of these public data sources. All datasets are accessible online through their respective repositories.
